# The Biological Roles of lncRNAs and Future Prospects in Clinical Application

**DOI:** 10.3390/diseases9010008

**Published:** 2021-01-13

**Authors:** Guohui Li, Liang Deng, Nan Huang, Fenyong Sun

**Affiliations:** 1 School of Life Sciences, Jiangsu University, 301# Xuefu Road, Zhenjiang 212013, China; ghli@ujs.edu.cn (G.L.); denglianghp@163.com (L.D.); 2 Department of Clinical Laboratory Medicine, Shanghai Tenth People’s Hospital of Tongji University, Shanghai 200072, China; 1532170@tongji.edu.cn

**Keywords:** lncRNA, DNA damage, genomic integrity, repair, cancer

## Abstract

Chemo and radiation therapies are the most commonly used therapies for cancer, but they can induce DNA damage, resulting in the apoptosis of host cells. DNA double-stranded breaks (DSBs) are the most lethal form of DNA damage in cells, which are constantly caused by a wide variety of genotoxic agents, both environmentally and endogenously. To maintain genomic integrity, eukaryotic organisms have developed a complex mechanism for the repair of DNA damage. Researches reported that many cellular long noncoding RNAs (lncRNAs) were involved in the response of DNA damage. The roles of lncRNAs in DNA damage response can be regulated by the dynamic modification of N6-adenosine methylation (m6A). The cellular accumulation of DNA damage can result in various diseases, including cancers. Additionally, lncRNAs also play roles in controlling the gene expression and regulation of autophagy, which are indirectly involved with individual development. The dysregulation of these functions can facilitate human tumorigenesis. In this review, we summarized the origin and overview function of lncRNAs and highlighted the roles of lncRNAs involved in the repair of DNA damage.

## 1. Introduction

DNA damage is constantly caused by various endogenous and exogenous factors, such as ionizing radiation, ultra-violet, reactive oxygen species (ROS), and genotoxic drugs [1,2]. It is generally accepted that DNA damage is a potential threat to human health. Human have evolved intricate mechanisms for the repair of DNA damage to sustain genome stability, and homologous recombination (HR) and nonhomologous end joining (NHEJ), as two major DSBs repair pathways, have been ubiquitously applied in cells [3,4]. If living organisms fail to accurately repair the damaged DNA in cells, the accumulation of DNA damage will lead to serious consequences and, eventually, the occurrence of cancers in the body. So, genomic integrity is essential for organism survival and for the inheritance of traits to offspring. Long noncoding RNAs (LncRNAs) are an important class of RNA transcripts, with over 200 nucleotides in length, which resemble protein-coding genes but lack the ability for translation into proteins in general [5]. Hangauer et al. [6] reported that over 10,000 lncRNA transcripts could be produced from the human genome, and some lncRNAs were reported to play regulatory roles in various biological processes, ranging from the innate immune response, cell cycle control, pluripotency, and differentiation to disease [7,8,9]. Moreover, recent evidence showed that some lncRNAs such as NORAD and GUARDIN could directly participate in the repair of DNA damage [9,10,11].

Different classes of lncRNAs were transcribed from several DNA elements, such as enhancers, promoters, and intergenic regions, in eukaryotic genomes [12]. Iyer et al. (2015) [13] reported that over 50,000 lncRNAs (designated MiTranscriptome lncRNAs) could be generated in the human transcriptome from various tumors, normal tissues, and cell lines based on The Cancer Genome Atlas (TCGA; http://cancergenome.nih.gov/). To date, 268,848 lncRNAs have been collected in the database of human lncRNAs (https://bigd.big.ac.cn/lncbook/index), which is far greater than the number of protein-coding mRNAs (~20,000) in the genome. Unlike protein-coding mRNAs, lncRNAs exhibit functional uniqueness by participating in and modulating various cellular processes, including histone modification, DNA methylation, cellular transcription, the inflammatory response, antiviral immunity, and repair of DNA damage [14,15,16,17]. Additionally, some lncRNAs also function as diagnostic markers and/or possible therapeutic targets. Therefore, the understanding of biogenesis and the biological functions of lncRNAs is helpful for disclosing their functional significance.

## 2. Biogenesis of lncRNAs in Eukaryotes

**Figure 1:**
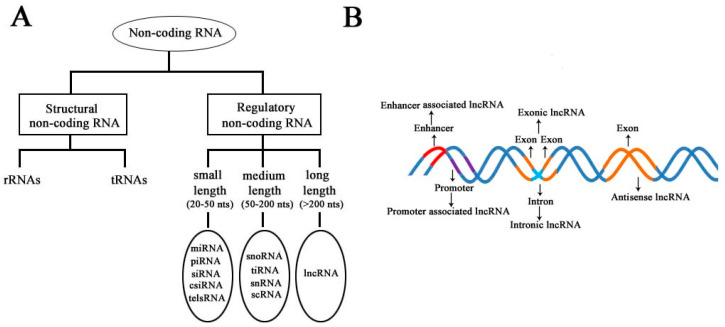
Schematic diagram to illustrate the diversity of long noncoding RNAs (lncRNAs) in the mammalian genome. (**A**) Classification of noncoding RNAs according to their size and function. (**B**) Overview of the biogenesis of various lncRNAs through different mechanisms. Different colors indicate different regions of DNA elements in the mammalian genome.

According to the diversity of noncoding RNAs, they can be divided into two main types: structural noncoding RNAs and regulatory noncoding RNAs [8]. Structural noncoding RNAs comprise of rRNAs and tRNAs, and regulatory noncoding RNAs are further divided into three classes: small, medium, and long noncoding RNAs (Figure 1A) [18,19]. The biogenesis of lncRNAs is cell type- and stage-specific, which is under the control of cell type- and stage-specific stimuli. Different classes of lncRNAs were reported to be transcribed from different DNA elements, such as enhancers, promoters, and intergenic regions, in eukaryotic genomes (Figure 1B). As we know, promoters and enhancers are essential DNA elements in the control of gene expression networks. Some short-lived medium-length lncRNAs can be transcribed from promoter upstream regions and enhancers by RNA polymerase II (Pol II), and some lncRNAs can be bidirectionally transcribed from enhancers by Pol II [20,21]. Additionally, some lncRNAs are transcribed by Pol II from intergenic regions between two genes and represent the best-studied subclass of lncRNAs.

Most annotated lncRNAs contain multiple exons and have typical mRNA-like features, with a 5′ m^7^G cap and a 3′ poly(A) tail. These similarities existing between lncRNAs and mRNAs provide the possibility that mature lncRNAs may behave similarly to mRNAs in cells. In fact, this is not the truth. Due to a lacking of robust protein-coding potential, lncRNAs are less evolutionarily conserved and less abundant. They exhibit more tissue-specific expression and greater nuclear localization patterns. Additionally, a significant difference was found among different lncRNAs varying in their sizes. In the database of lncRNA (http://lncrnamap.mbc.nctu.edu.tw/php), the statistics of the lncRNA classes show that there are 23,879 lncRNAs with length <1000 nt, 4985 lncRNAs with lengths ranging from 1000 to 2000 nt, 1943 lncRNAs with lengths ranging from 2000 to 3000 nt, and 12 lncRNAs with lengths ranging from 9000 to 10,000 nt). Moreover, some lncRNAs were found to be involved in the DNA damage response, which are summarized in Table 1.

## 3. Involvement in a Variety of Biological Functions

LncRNAs are characterized by the wide diversity, which is consistent with their diverse roles in a wide variety of biological processes. LncRNAs can regulate gene expressions at the levels of transcription and translation. Additionally, lncRNAs also participate in several aspects of DNA damage response and genomic stability maintenance.

### 3.1. LncRNAs Participating in Transcription Regulation

Increasing evidence indicates that lncRNAs can function as transcriptional regulators. Numerous lncRNAs have been reported to function in many cases as transcriptional regulators, which can bind with different partners to exert their functions. Some lncRNAs can bind to transcription factors and RNA polymerase II for the transcriptional regulation of various genes. This action can be either in cis or in trans at the transcriptional level. For example, PANDAR is known to interact with the nuclear transcription factor Y subunit A (NF-YA), which can inhibit the expression of apoptotic genes [31]. Furthermore, Pospiech et al. [32] reported that PTBP1 could interact with PANDAR, and the interaction was confirmed to be involved in splicing regulation. Miao et al. [33] reported that the lncRNA LEENE could facilitate the recruitment of RNA Pol II to the eNOS promoter to enhance the eNOS nascent RNA transcription.

LncRNAs can be located within cellular compartments such as the nucleus, nucleolus, and cytoplasm. Six thousand, seven hundred and sixty-eight GENCODE-annotated lncRNAs across various compartments of 15 cell lines are collected in the database of lncATLAS (http://lncatlas.crg.eu/). Of these, 31 lncRNAs can be detected in all samples tested, and lncRNAs display a highly cell type-specific expression pattern [34]. The cellular localizations of lncRNAs are indicative of their functions. Researches reported that most lncRNAs were located in the nucleus and performed their functions through forming complexes. For example, the lncRNA of Kcnq1ot1 is localized exclusively in the nuclear compartment, which can interact with the G9a of a histone methyltransferase to facilitate the transcriptional silencing of target genes related to the development of mouse placenta [35,36]. Some lncRNAs such as TUG1 and MALAT1/NEAT2 can bind with CBX4 to stimulate the sumoylation of the E2F1 growth factor, which can lead to activation of the growth control gene [37]. Compared with nuclear lncRNAs, cytoplasmic lncRNAs are less well-understood. Now, accumulating evidence indicates that cytoplasmic lncRNAs can form complexes with diverse structural and regulatory functions. For example, NORAD is an abundant 5.3-kb unspliced polyadenylated transcript that localizes predominantly in the cytoplasm, and it can interact with PUMILIO proteins to facilitate the stability and translation of mRNAs for the maintenance of genomic stability [38]. Lu et al. [39] reported that lncRNA-DANCR could modulate mTOR expression by sponging miR-496 to facilitate the progression of lung adenocarcinoma. Moreover, several p53-induced lncRNAs such as TUG1 and PINT1 are involved in negatively regulating p53 targets [40,41].

Furthermore, lncRNAs also can modulate the expression of target genes at the level of post-transcription. For example, Gonzalez et al. [42] reported that an evolutionarily conserved nuclear antisense lncRNA could promote the epithelial-specific alternative splicing of FGFR2 pre-mRNA. Additionally, several studies identified the involvement of oncogenic nuclear lncRNA MALAT1 in alternative splicing regulation [43,44]. The lncRNAs are summarized in Table 2. In a word, lncRNAs can regulate gene expressions via multiple diverse mechanisms. However, the dysregulation may be directly involved with individual development and tumorigenesis [45,46].

### 3.2. Involvement of lncRNAs in the Repair of DNA Damage

Several types of DNA damage, including single-stranded break, double-stranded break, base mismatches, bulky adducts, and base alkylation, may be produced constantly in the host cell [47]. The cellular damage will amplify the cascade signal, leading to cell death or cancerization if they cannot be repaired immediately. Meanwhile, cells have evolved the ability to repair the lesion and maintain genome integrity when the genome of the host cell gets damaged. Although various lncRNAs were involved in the repair of DNA damage, the underlying mechanism of DNA repair behind the phenomenon remains insufficiently understood [48].

Many factors, including RNA-binding proteins and lncRNAs, can be recruited at DNA damage sites, indicating that they may have important roles during the response of DNA damage. To date, various lncRNAs have been shown to participate in the repair of DNA damage, and these lncRNAs usually exert their functions via interactions with protein complexes [48]. For example, Sharma and colleagues identified lncRNA DDSR1 as a regulator of DNA repair by homologous recombination [27]. DINO, a conserved DNA damage-inducible lncRNA, was identified as a new component for the stability of p53 and the regulator of the p53-dependent DNA damage response [49]. LncRNAs CUPID1 and CUPID2 were predominantly expressed in hormone receptor-positive breast tumors, which can modulate the repair of double-stranded breaks by the NHEJ and HR pathways [50]. Jiao et al. [51] reported that X-ray-inducible LIRR1 with a 273-bp length could regulate DNA damage response signaling in the human bronchial epithelial BEAS-2B cell line. While the HR pathway requires a homologous template, the NHEJ pathway repairs DSBs by directly ligating the ends. To better understand the roles of lncRNAs in the repair of DNA damage, the involvement of various lncRNAs in the repair of DNA damage are summarized in Table 3.

### 3.3. Clinical Biomarkers in Cancer Patients

Mounting evidence suggests that the dysfunction of lncRNAs is implicated in a wide variety of diseases, especially with cancer, and the distinct expression profiles of lncRNAs are often used as biomarkers for disease types and stages [55,56,57]. LncRNA disease 2.0 is freely available at http://www.rnanut.net/lncrnadisease/, in which more than 200,000 lncRNA–disease associations are collected. As of October 2020, a search in the National Institutes of Health PubMed database with the keywords “long noncoding RNA” and “Cancer” produced >14,126 publications. These lncRNAs can regulate various aspects of cellular homeostasis, including the survival, proliferation, invasion, metastasis, and angiogenesis of cancer cells. Furthermore, some lncRNAs can function as oncogenes or tumor suppressors, and they were further summarized in a list by Chandra and Nandan [58].

HOTAIR and FAL1 can function as oncogenes, which are directly involved with the occurrence and development of cancers [59,60,61]. HOTAIR was reported to be frequently upregulated in various types of cancer, including breast cancer, esophageal cancer, lung cancer, gastric cancer, and melanoma [60,62]. Moreover, HOTAIR was further developed as a diagnostic marker for lymph node metastasis [63]. Additionally, lncRNA small nucleolar RNA host gene 1 (SNHG1) also functions as an oncogene in various human cancers [64]. Some lncRNAs were often found to be dysregulated in various of cancers, which were potentially used as biomarkers in the diagnosis of cancer [65,66]. For example, MALAT1 showed a marked upregulation in lung cancer, breast cancer, colorectal cancer, bladder carcinoma, and hepatocellular carcinoma [67]. Therefore, it could be used as a potential biomarker for the early diagnosis of cancer, as well as prognosis.

As is well-known, some cancers have entered the middle and late stages when they are discovered. Therefore, the early diagnosis of cancer in patients is key to providing personalized treatment strategies, and it is promising to improve the clinical outcome. To this purpose, some lncRNAs have been developed to be novel diagnostic markers for cancer. For example, the lncRNA PCA3 was firstly developed as a routine biomarker for the diagnosis of prostate cancer [68]. Yuan et al. [69] reported that the three lncRNAs of LINC00152, RP11-160H22.5, and XLOC014172 may function as novel biomarkers for diagnosis of HCC patients. Recently, lncRNA-D16366 has been reported to be a potential biomarker for the diagnosis and prognosis of HCC [70]. They are summarized in Table 4.

### 3.4. Regulation of Autophagy by lncRNAs

Various cellular lncRNAs can regulate autophagy, which is a highly conserved cellular process to maintain the homeostasis in eukaryotes [71]. The dysfunction of autophagy can cause the pathogenesis of numerous human diseases, including cancers. So far, the three critical protein complexes include the ULK1-ATG13-FIP200-ATG101 complex, the Beclin1-ATG14-Vps34-Vps15 (class III PI3-kinase) complex, and the ATG12–ATG5-ATG16L1 complex, and these complexes have been reported to be involved in the formation of autophagosomes [72]. Mounting evidence has shown that many proteins important for autophagy could be regulated by lncRNAs [73,74,75]. Now, studies for demonstrating the relationship between lncRNAs and autophagy are becoming a worldwide hot spot of life science.

The progression of some cancers can be achieved through the lncRNA-mediated regulation of autophagy. For example, Yang et al. [76] reported that HOTAIR could activate autophagy through the upregulation of ATG3 and ATG7 to facilitate the proliferation of hepatocellular carcinoma. The knockdown of HOTAIR could result in the silencing of miR-454-3p, which, subsequently, resulted in the reduction of a signaling cascade to the target, ATG12. It also further decreased autophagy in a chondrosarcoma cell line [77]. MALAT1, a well-established lncRNA, also promotes cancer proliferation and metastasis via the stimulation of autophagy. For example, MALAT1 could activate autophagy by sponging miR-101 and upregulating STMN1, RAB5A, and ATG4D expressions in the glioma and, also, modulate the autophagy of retinoblastoma cell through miR-124-mediated stx17 regulation [78,79]. Moreover, Malat1 was also involved with chemoresistance in gastric cancer and multidrug resistance in hepatocellular carcinoma cells via the modulation of autophagy [80,81]. They are summarized in Table 5. Therefore, it is important to elucidate the cellular mechanism of lncRNA for the regulation of autophagy. Furthermore, it can provide a novel strategy of prevention and treatment of tumors by the way of lncRNA-regulated autophagy.

According to the above reports, lncRNAs are regarded as functional transcripts that directly link with the occurrence and development of cancer. Therefore, they are becoming effective biomarkers of diagnosis and attractive potential therapeutic targets.

## 4. Expression Level of lncRNAs Regulated by m6A

N6-adenosine methylation (m6A) is the most common internal modification in mRNA and long noncoding RNA, and it is also a dynamic reversible modification with implications in fine-tuning the cellular metabolism. It is modulated by m6A regulators, including “writers” (methyltransferases), “readers” (signal transducers), and “erasers” (demethylases) [82]. To date, this modification has been identified in various organisms, including yeasts, plants, flies, mammals, and some viruses, and more than 12,000 m6A sites in the transcripts of ∼7000 protein-coding genes and ∼300 noncoding genes have been characterized in human cells [83]. Furthermore, the majority of m6A was found within the conserved RRACH motif (R = G/A and H = A/C/U) in mRNAs. Meanwhile, many lncRNAs could be modified by m6A, which can control many aspects of gene expression and cellular biology at both the transcriptional and post-transcriptional levels [84,85,86].

However, the aberrant expression and dysregulation of lncRNA is strongly linked to tumorigenesis, metastasis, and the tumor stage [87,88]. For example, MEG3 and NBAT1 have been confirmed to play an important role in the formation of pathogenicity of gliomas [89,90]. Moreover, MALAT1 is highly expressed in the nucleus, and it has been confirmed to play a suppressive role in the formation of gliomas by downregulating MMP2 and devitalizing ERK/MAPK signaling [91]. The m6A modification has been confirmed to play functional roles in RNA splicing, nuclear export, and decay [92]. For example, the MALAT1 with m6A modification could regulate the interaction between RNAs and some special binding proteins and, also, affect its localization and activity in the nucleus [93]. Now, the m6A modification has been identified as the most abundant modification in mRNA and noncoding RNA (ncRNA). Accumulating studies have focused on the role of lncRNAs regulated by m6A modification in cancer progression, and it was used to demonstrate the mechanisms by which m6A participates in the biology of cancers.

**Figure 2:**
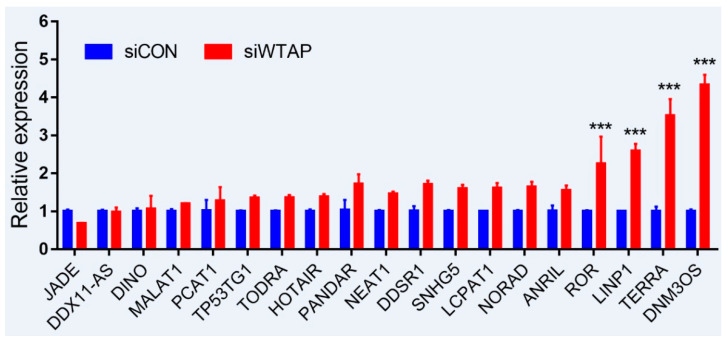
Expression analysis of lncRNAs through RT-qPCR from the siControl- and siWTAP-transfected HCC cell lines (SMCC7721). The error bar represents the standard deviation of each mean value (mean ± SD; *n* = 3). Asterisks indicate significant differences compared with the controls. *** represents significant difference compared with control group.

As is well-known, DNA damage is closely involved with the occurrence and development of cancers, and many lncRNAs were involved in the repair of DNA damage [94,95]. To further examine whether the expression of lncRNAs is regulated by m6A, an analysis of RT-qPCR was performed to detect the expressions of some lncRNAs related to DNA damage. For the purpose, some lncRNAs from siControl- and siWTAP-transfected HCC cell lines (SMCC7721) were selected for the analysis. The results showed that the expressions of ROR, LINP1, TERRA, and DNM3OS were significantly increased by over two-fold compared with the control group (Figure 2, unpublished data). Additionally, the expressions of DDSR1, SNHG5, LCPAT1, NORAD, and ANRIL also showed a 1.5-fold increase compared with the control group, and the statistical analysis further showed that there were significant differences between siControl and siWTAP (Figure 2). As far as the rest of the lncRNAs (Figure 2) were concerned, no obvious changes in the expression levels were observed compared with the control group. Therefore, we think that the expressions of some lncRNAs related to DNA damage could be regulated by m6A, indicating that the modification may play an important role in the regulation of the DNA damage response. However, abnormal regulation may directly promote tumorigenesis.

## 5. Future Prospects

The early detection of cancers is very critical for preventing the occurrence and development of metastatic diseases. Although some proteins have already been applied for the detection of tumors, their sensitivity and clinical staging abilities are not favorable for the treatment of cancers. It can be disastrous for cancer patients if a diagnosis is made during the middle and late stages of cancer. Therefore, the development of novel biomarkers can not only facilitate the early detection of cancers but, also, improve the physical health of tumor patients.

LncRNAs were once considered as dark matter and junk DNA for decades because of a bunch of evidence for their failure to encode proteins [96]. Now, mounting evidence indicates that lncRNAs participate in various biological processes, including the occurrence and development of cancers. Furthermore, some lncRNAs were found to be regulated by m6A modification (Figure 2), which is closely involved in the repair of DNA damage. If lncRNAs are dysregulated by some factors such as the m6A modification, they may result in cellular imbalances, including DNA damage. Furthermore, the accumulation of DNA damage leads to diseases, even cancers. The emerging roles of lncRNAs in the development of human cancers are diverse, and the mechanisms of lncRNAs in tumorigenesis are very complex. Further researches are required to demonstrate the correlations between lncRNAs and cancers.

In conclusion, the changes of the expression patterns of some specific lncRNAs can be indicative of cancers. We think that lncRNAs may be promising diagnostic biomarkers for the detection of cancers, and they can be utilized to predict the prognosis of cancer patients in the future.

## Figures and Tables

**Table 1:** Different long noncoding RNA (lncRNA) involvement with DNA damage.

LncRNAs	Accession Number	Functions	Length (nts)	Genome	Refs.
NORAD	NR_027451.1	Critical for genome stability	5378	Human	[10]
GUARDIN	NR_132738.1	Critical for genome stability	1003	Human	[11]
LCPAT1	NM_018715.4	Involvement with DNA damage	4040	Human	[17]
LINC00261	NR_001558.3	Activation of DDR	4924	Human	[21]
Meg3	NR_046473.1	Regulation of DNA damage response	9701	Human	[22]
DNM3OS	NR_038397.2	Regulation of DNA damage response	7957	Human	[23]
LINP1	NR_138480.1	facilitating DNA damage repair	838	Human	[24]
DINO	NR_144384.1	Efficient activation of p53 target genes	951	Human	[25]
TODRA	NR_040058.1	Promoting RAD51-dependent DSB repair	1156	Human	[26]
DDSR1	KT318134.1	Modulating DNA repair by HR	1616	Human	[27]
JADE	KC469579.1	Functional linking with histone H4 acetylation	1721	Human	[28]
NEAT1	MK562403.1	A common mediator for inflammasome stimuli	2713	Human	[29]
ROR	HG975412.1	A p53 repressor in response to DNA damage	2591	Human	[30]

Note. DDR and HR are the abbreviations for DNA damage response and homologous recombination. DSB: double-stranded break. Refs is the abbreviations for references.

**Table 2:** LncRNA functions in transcription regulation.

LncRNAs	Localization	Potential Targets	Functions	Refs.
PANDAR	Nuclear	NF-YA	Inhibition of apoptotic genes expression	[31]
PANDAR	Nuclear	PTBP1	Splicing regulation	[32]
LEENE	Nuclear	Recruitment of RNAPⅡ to the promoter	Enhancement of eNOS transcription	[33]
Kcnq1ot1	Nuclear	G9a of histone methyltransferase	Silence of genes related to mouse placenta development	[35]
TUG1	Nuclear	CBX4 and E2F1 sumoylation	Activation of growth control genes	[37]
MALAT1/NEAT2	Nuclear	CBX4 and E2F1 sumoylation	Activation of growth control genes	[37]
NORAD	Cytoplasm	PUMILIO	Maintenance of genome stability	[38]
DANCR	Cytoplasm	miR-496	Modulation of mTOR expression	[39]
PINT1/TUG1	Cytoplasm	P53	Negatively regulation of p53 targets	[40,41]
MALAT1	Nuclear	Unknown	Alternative splicing regulation	[43,44]

**Table 3:** LncRNAs involved in DNA damage/repair. NHEJ: nonhomologous end joining.

lncRNA	Localization	Potential Targets	Roles	Refs.
NORAD	Cytoplasm	TOP1, RBMX, UMILIO	Contribution of maintaining genomic stability	[9,10]
LINC00261	Nucleus	ATM kinase, TOP2A	Activation of the DNA damage response	[21]
Meg3	Nucleus	Mdm2, PTBP3	Regulation of DNA damage response	[22]
DNM3OS	Exosome	PDGFβ/PDGFRβ/FOXO1	Regulation of DNA Damage Response	[23]
LINP1	Cytoplasm	Ku80, DNA-PKcs	Facilitation of DNA damage repair by NHEJ pathway	[24]
TODRA	Nucleus	RAD51	Enhancement of RAD51-dependent DSB repair	[26]
DDSR1	Nucleus	BRCA1 and hnRNPUL1	Modulation of DNA repair by HR	[27]
JADE	Cytoplasm	Histone H4	Induction of histone H4 acetylation in the DDR	[28]
DINO	Nucleus	RRM2b, DDB2	Regulation of p53-dependent DNA damage response	[49]
CUPID1	Nucleus	Phosphorylated RPA	Modulation of the Response to DNA Damage	[50]
LIRR1	Nucleus	KU70, KU80, and RAD50	Mediation of DDR and DNA damage repair.	[51]
TP53TG1	Cytoplasm	PI3K/AKT signal pathways	Contribution of the p53 response to DNA damage	[52]
ERIC	Cytoplasm	E2F1, E2F3	Modulation of the cellular response to DNA damage	[53]
TERRA	Nucleus	TRF2, Suv39h1, ORC1	Telomere maintenance and genome stability	[54]

**Table 4:** LncRNAs as clinical biomarkers.

lncRNA	Function	Cancer Type	Refs.
HOTAIR	Oncogene	Breast Cancer, Esophageal Cancer, Lung Cancer, Gastric Cancer, and Melanoma	[60,62]
MALAT1	Oncogene	Lung Cancer, Breast Cancer, Colorectal Cancer, Bladder Carcinoma, and Hepatocellular Carcinoma	[67]
PCA3	Diagnosis	Prostate Cancer	[68]
LINC00152	Diagnosis	Hepatocellular Carcinoma	[69]
RP11-160H22.5	Diagnosis	Hepatocellular Carcinoma	[69]
XLOC014172	Diagnosis	Hepatocellular Carcinoma	[69]
lncRNA-D16366	Diagnosis and prognosis	Hepatocellular Carcinoma	[70]

**Table 5:** Regulation of autophagy by lncRNAs.

lncRNA	Mechanism	Function	Refs.
HOTAIR	Upregulation of ATG3 and ATG7	Facilitation of Hepatocellular Carcinoma proliferation	[76]
HOTAIR	Protect miR-454-3p from silencing	Increase of ATG12 and autophagy in a chondrosarcoma cell line	[77]
MALAT1	Sponge of miR-101 and upregulation of STMN1, RAB5A, and ATG4D expression	Autophagy activation in glioma	[78]
MALAT1	MiR-124-mediated stx17 regulation	Autophagy modulation of retinoblastoma cell	[79]

## Data Availability

Not applicable.
